# Towards an inclusive conference experience: evaluation of the Education and Outreach Symposium at the Microbiology Society Annual Conference 2024

**DOI:** 10.1099/acmi.0.000995.v3

**Published:** 2025-03-11

**Authors:** Melissa M. Lacey, Michael J. Dillon, Sean Goodman, Victoria Easton, Alison I. Graham

**Affiliations:** 1 School of Biosciences and Chemistry, Sheffield Hallam University, Sheffield, UK; 2 Peninsula Medical School, University of Plymouth, Plymouth, UK; 3 School of Dentistry, University of Liverpool, Liverpool, UK; 4 School of Molecular and Cellular Biology, University of Leeds, Leeds, UK; 5 School of Medicine, Newcastle University, Newcastle-upon-Tyne, UK

**Keywords:** conference, education, inclusive practice, outreach, pedagogy, public engagement

## Abstract

The Microbiology Society Education and Outreach Symposium serves as a platform for microbiology educators to share contemporary practices with an international audience. The Symposium is held yearly during the Microbiology Society’s Annual Conference and has become increasingly popular among conference attendees. In an effort to create an inclusive and engaging environment, the 2024 Symposium included contributions from participants at all career stages and from diverse global regions through a variety of presentation formats, including invited talks, offered presentations, flash talks and posters. Cabaret-style seating was used to encourage discussion amongst participants, and digital tools were used for anonymous feedback and questions after each talk to ensure all voices had an opportunity to be heard. Here, we present an analysis of qualitative and quantitative participant responses addressing two key research questions: (1) Did the Symposium foster an inclusive atmosphere for participants across all career stages? and (2) Was the content engaging and relevant to the audience? A post-Symposium questionnaire revealed strong positive feedback, with all 18 respondents agreeing or strongly agreeing that the 2024 Symposium was both an inclusive environment and covered interesting topics. Thematic content analysis of free-text responses emphasized a high appreciation for the Symposium’s diversity in speakers and topics, an inclusive room layout and an overall welcoming feeling. Feedback from participants, along with the authors’ own reflections, will actively feed into planning for the 2025 Symposium.

## Data Summary

Data used to generate results can be accessed at FigShare [1].

## Introduction

The Education and Outreach Network (EON) [2] is a member-led group within the Microbiology Society that supports colleagues in their efforts to educate and inform diverse audiences about microbiology. EON facilitates the exchange of ideas and best practices among educators through sponsored workshops, links with *Access Microbiology* [3] and through the Education and Outreach Symposium at the Microbiology Society’s Annual Conference [4,5].

The Education and Outreach Symposium was first held as a pre-conference session to the Annual Conference in 2018 [6]. It has since become a parallel session alongside the scientific sessions, where it is the platform for colleagues to share contemporary microbiology education and outreach work with an international audience of peers. The 2024 Symposium showcased contributions from participants at all career stages and from globally diverse geographical locations. There was a mixture of 30-min invited presentations, 15-min offered talks, 5-min flash talks and posters [5]. All of the flash talks had an accompanying poster to encourage continued engagement with the presenter. The Symposium consisted of two sessions, Wednesday afternoon and Thursday morning, with attendance ranging from 40 to 93 participants.

In our continued effort to ensure the Symposium is welcoming and inclusive, we collected quantitative and qualitative data from participants. This initiative represents a shift towards a more evidence-based approach to evaluating the Symposium’s impact and effectiveness. Specifically, this article seeks to address two questions:

Research Question 1 - Inclusivity: does the Symposium effectively welcome a diverse audience across all career stages?

Research Question 2 - Relevance: is the content provided interesting to participants?

This article will first present a synopsis of the Symposium organized as topics. Second, it will present a detailed analysis of data collected to address the research questions above, highlight key themes, and offer insights from those who organized and chaired the sessions. By sharing our findings, we hope to contribute to the broader conversation on effective strategies for inclusive and relevant microbiology education and outreach.

## Synopsis of the Education and Outreach Symposium

### Artificial intelligence

Artificial intelligence (AI) burst onto the educational landscape with the emergence of large language models, such as ChatGPT 3.5, allowing the user to produce written outputs as well as undertake data analysis, coding and image analysis [7]. Pam Birtill [8] opened the Symposium by sharing the current state of play and up-to-date research around the ‘double-edged sword’ that is the use of AI (specifically large language models) in higher education (HE) assessment. Gayan Gunatilake continued the AI-in-assessment theme by discussing the efficacy of ChatGPT responses to bacterial species-specific questions. Their research emphasized the importance of question formulation on the accuracy of answers. Catherine Lawler shared how they had used AI to encourage students to engage in self-evaluation. Students were invited to evaluate essays, some of which had been generated by ChatGPT 3.5. Using the innovative metric of ‘edits per minute’, they determined that students were 53% more critical of essays they knew to be AI-generated. Niall O'Leary then showed how field trips for microbiology undergraduates could be redefined by combining 360° mapping with AI manipulation.

### Sustainability

The United Nations (UN) Sustainable Development Goals are a set of 17 wide-ranging targets to tackle climate change, preserve biodiversity and support equitable human development [9]. All UN members have adopted these goals within the 2030 Agenda for Sustainable Development [10]. Zoe Robinson [11] spoke passionately about our shared responsibility for sustainability within HE and how we can embolden our students to become global citizens, able to create a sustainable future. Following this, Lizzie Archer shared their microbiology/sustainability outreach practice, where they embedded green spaces ('tiny forests') at a local primary school to provide opportunities for pupils to interact with nature and discuss the microbiology of the woodland ecosystem. This led to the development of an educator toolkit. Linda Percy described co-creating student research projects using a marine bacteria culture collection linked to sustainability.

### Innovations in higher education

Discussions around AI and sustainability set the scene for the broader topic of innovations in HE. Leighann Sherry and Gemma Wattret both focused on employability skills. Leighann Sherry shared how they had used LinkedIn to increase undergraduate students’ career awareness and confidence in networking [12]. Gemma Wattret explained how they had used an authentic interdisciplinary enterprise challenge to address the employment attainment gap for Black, Asian and minority ethnic students by increasing employability skills, widening professional networks and raising awareness of career opportunities.

Three presentations concentrated on skills in the laboratory. Rebbekah Menday shared their work on enhancing digital accessibility in taught laboratory sessions by using assistive technologies. The pressured nature of laboratory classes can be off-putting for some students; Andy Gilbert described an extra-curricular laboratory skills development programme that aimed to enhance student confidence and competence in a relaxed environment. At the same time, laboratory classes can be a key driver in building a student community, as Victoria Easton explained.

Innovative technologies were also highlighted by Bridget Kelly, who described how to use Photovoice to engage undergraduate students in microbiology, and Arindam Mitra, who presented a new system for proctoring of online exams for use with higher-order thinking questions.

### Outreach

Public engagement in microbiology, often referred to as outreach, was a core theme of the Symposium. Several presenters shared their outreach practices aligned with the Microbiology Society initiative ‘Knocking Out AMR (antimicrobial resistance)’ [13]. Anderson Oaikhena presented an inspiring talk on their ambitious project that aimed to increase AMR awareness in Nigeria by equipping postgraduate students to act as ‘anti-AMR advocates’.

A number of presenters described how they formed interdisciplinary collaborations to share important AMR messages with diverse audiences: Mark Erickson blended AMR and classical Greek theatre [14], Ali Floyd’s team looked at tuberculosis through a local history lens [15], Leanne Timpson brought science and games art students together and Maria del Carmen Montero-Calasanz worked with science and humanities students to decide on names for new bacterial species [16]. In addition, Moe Kyaw Thu engaged with Malaysian school children using a multifaceted approach around microbiology, and Emma Waters discussed their ‘What is a scientist?’ project, aimed at smashing stereotypes in science.

**Fig. 1.**
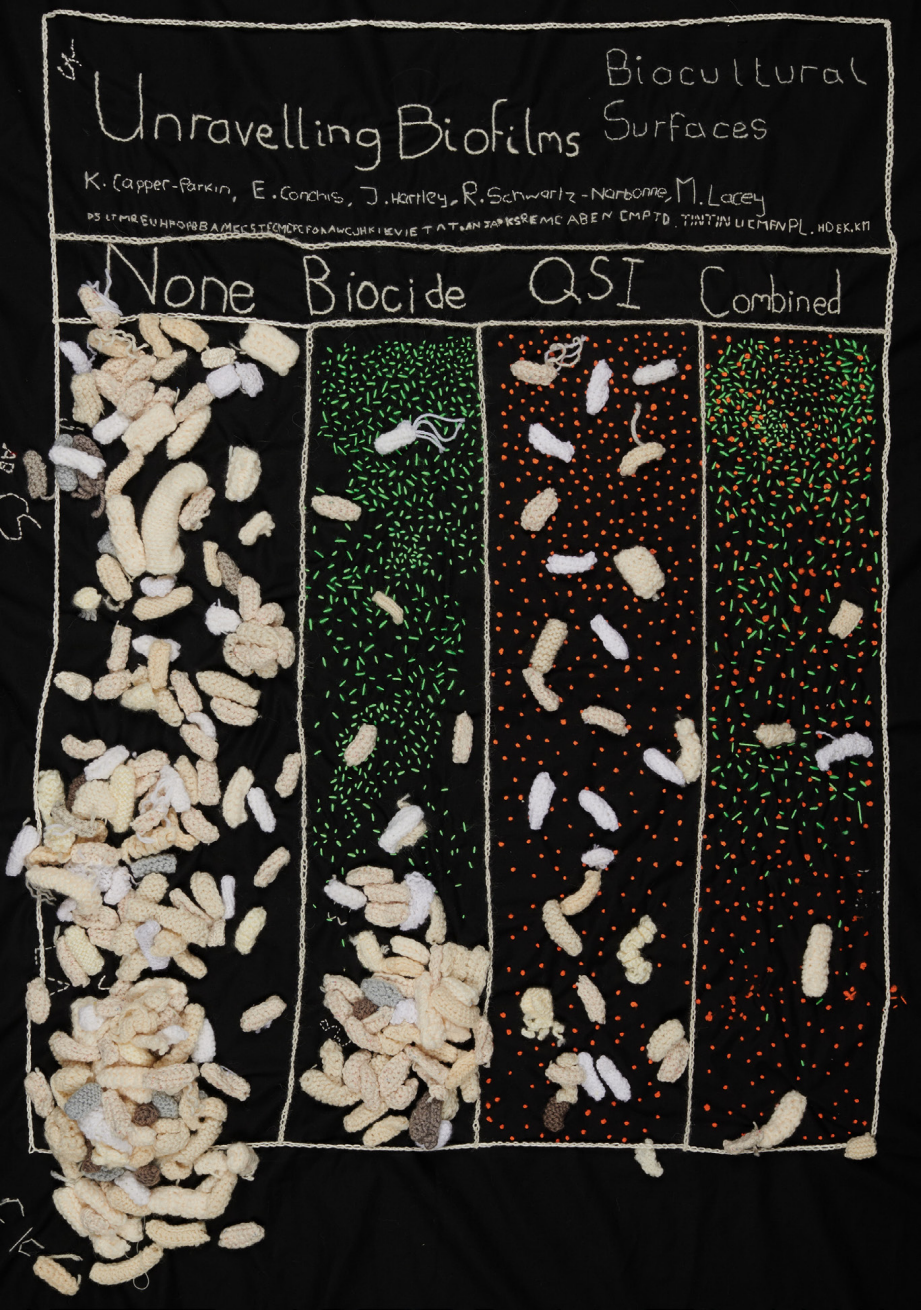
‘Unravelling Biofilms: Biocultural Surfaces’. *Access Microbiology* poster prize winner submitted by Kelly Capper-Parkin. The poster was created with community groups, who used yarn-based crafts, such as knitting and crocheting, while engaging in discussions related to Kelly’s PhD research [29]. Image reproduced with the permission of Kelly Capper-Parkin [30].

As well as verbal presentations, there were also a wide variety of projects displayed in poster form (e.g. [17]). *Access Microbiolog*y, the open research journal of the Microbiology Society, gave poster prizes to Kelly Capper-Parkin for her innovative yarn-based poster on biofilms (Fig. 1) and Lizzie Archer for her project on ‘tiny forests’ (described above).

This section has provided an overview of the Symposium; abstracts for all presentations are available on the Microbiology Society’s website [5]. We now evaluate the Symposium.

## Methods

### Questionnaire design

A questionnaire was designed to determine if the Symposium provided an inclusive environment and covered interesting topics. Attendees of the Symposium were invited to take part in the questionnaire before each break and at the end of each half-day session. Participants accessed the online questionnaire hosted in Google Forms by following a QR code or shortened URL. These were shown on a presentation slide and were available on posters within the Symposium venue.

Three questions were used: (i) ‘I feel the 2024 Education and Outreach Symposium was an inclusive environment’ and (ii) ‘I feel the 2024 Education and Outreach Symposium covered interesting topics’. These questions both had Likert-style answer opinions of ‘strongly disagree’, ‘disagree’, ‘agree’, ‘strongly agree’ followed by a free-text response of ‘Please explain your answer’. The third question (iii) ‘What changes would improve future Education and Outreach symposia at the Microbiology Society Annual Conference?’ was a free-text response and was included to further improve the Symposium in 2025.

### Thematic content analysis

Thematic content analysis was performed [18] to identify patterns or themes within the free-text data from the three questions.

### Ethical approval

Ethical approval for this study was obtained from the School of Health, Wellbeing and Life Sciences Ethics Committee at Sheffield Hallam University, following the Sheffield Hallam University Research Ethics Policy. Ethical approval was given (reference: ER65407826). No identifiable, confidential or controversial information was collected. No gender, age, educational experience or other demographic factors were requested or considered within the analysis, primarily to ensure the questionnaire was concise and that length was not a barrier to completion. Participation in the study was optional, as stated in the online questionnaire, with opt-in consent being obtained.

## Results

### Embedding an inclusive culture

**Fig. 2.**
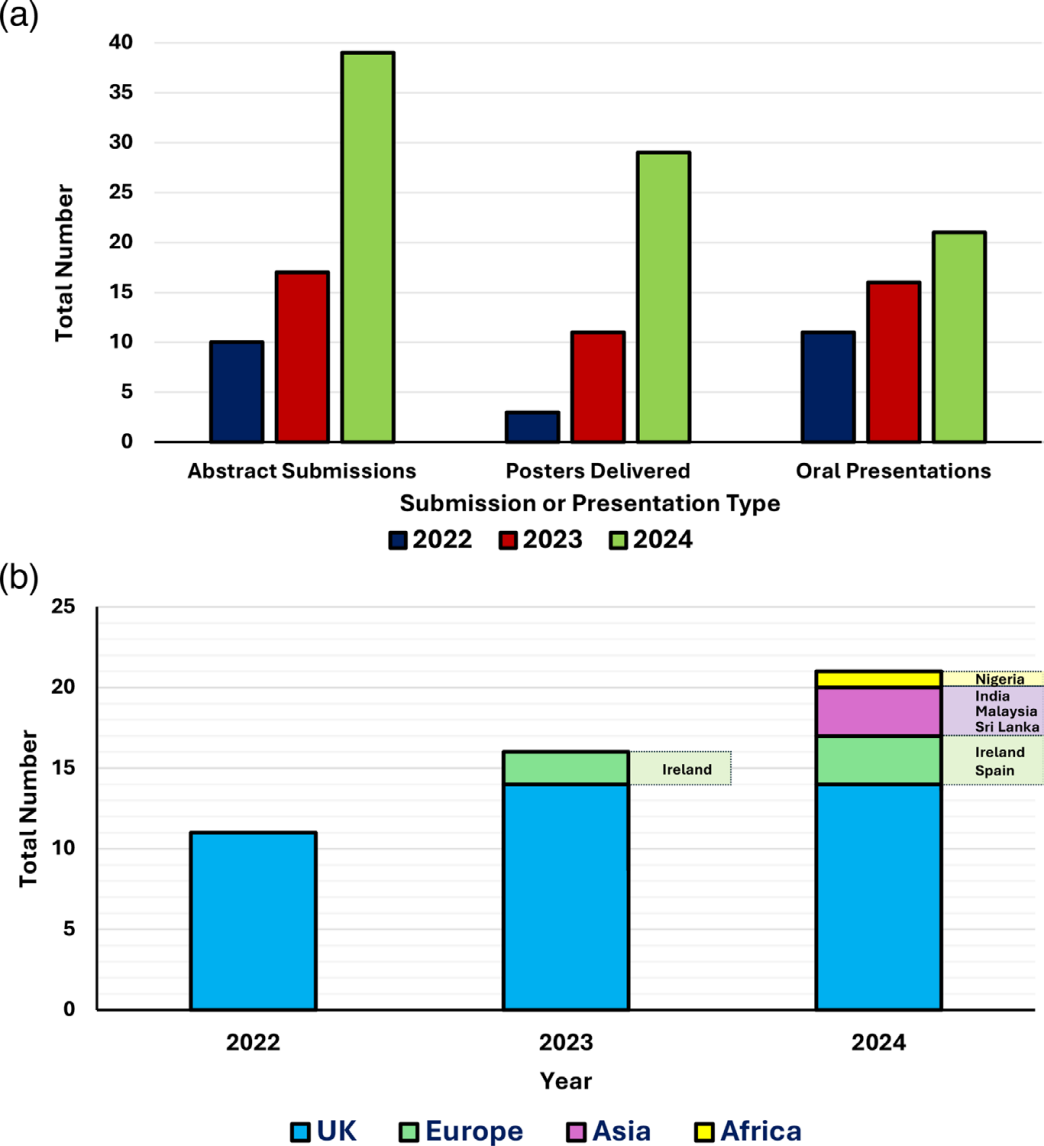
Expanding reach and diversity in submissions and presentations at the Education and Outreach Symposium (2022–2024). (a) Growth in submissions and presentations. Total numbers of abstract submissions and delivered posters and oral presentations. ‘Abstract submissions’ includes requests for both poster and oral presentations. Submissions could be accepted to present as both a poster and 5-min flash presentation. ‘Oral Presentations’ includes five invited talks in 2022, four invited talks in 2023 and three invited talks in 2024, which are not included in the total number of abstract submissions, due to the nature of these being invited by the organizing committee. (b) Global representation of offered talks. Geographical diversity in presentations (15-min offered talks and 5-min flash talks) delivered.

The Symposium has evolved significantly, growing from a pre-conference satellite session to an essential forum for sharing pedagogic research in microbiology teaching and outreach. For the 2024 Symposium, 39 abstract submissions were received, an increase from previous years (17 in 2023, ten in 2022 and seven in 2021) (Fig. 2a), underscoring the importance and increasing visibility of the Symposium. Microbiology Society members are vocal about supporting the diverse microbiology community, emphasizing the importance of diverse perspectives, and we actively encourage participation from all individuals [19,21].

For the 2024 Symposium, we took deliberate steps to ensure a diverse range of speakers and participants, reflecting various backgrounds, genders and career stages, as it is well-documented that role models play key roles in establishing a sense of belonging in members of under-represented groups [22,26]. The session featured three invited presentations, six offered presentations, 12 flash talks and 29 posters. Notably, seven of the 21 presentations (33%) were delivered by international speakers travelling from India, Ireland, Malaysia, Nigeria, Spain and Sri Lanka (Fig. 2b). Our goal was to recognize the diverse microbiology community and avoid tokenism, striving instead for genuine inclusion.

The programme was arranged so there was time for open-floor questions and discussions after each talk. Active participation was encouraged throughout the sessions by creating a safe and inclusive environment. For example, we were very intentional about our seating arrangements, using cabaret-style seating to facilitate networking and smaller discussions [27]. It is known that some demographic groups are more comfortable asking questions at conferences than others [28], and so we used anonymized digital tools (e.g.Padlet) to allow attendees to submit questions and feedback to be read out by the session chairs straight from their device. Flip charts captured anonymous feedback from those more comfortable with traditional formats. In these ways, participants could engage with the whole Symposium, engage at their table, and even engage anonymously, with the intention of allowing everyone to feel valued and heard.

### Analysis of delegate feedback

Attendees were invited to participate in a questionnaire about their experiences of the Education and Outreach Symposium. The 18 respondents to the questionnaire all ‘strongly agreed’ (15 responses, 83%) or ‘agreed’ (three responses, 17%) with the statement that the 2024 Education and Outreach Symposium was an inclusive environment. Similarly, all respondents ‘strongly agreed’ (14 responses, 78%) or ‘agreed’ (four responses, 22%) with the statement that the 2024 Education and Outreach Symposium covered interesting topics.

Forty free-text responses were received from the 18 respondents, resulting in the generation of three themes from seven coded response types (Table 1).

**Table 1.** Thematic content analysis of free-text responses (*n* = 40). Numbers in brackets indicate the number of responses received

Themes	Coded responses
Diversity (26)	Topic (14)
	Speaker (9)
	Presentation length (3)
Environment (7)	Positive atmosphere (4)
	Room layout (3)
Improvements (7)	Scheduling (5)
	Presentation length (2)

Diversity was the most frequently coded response type with 26 responses. Symposium attendees said they felt positive about the diversity of speakers, the oral presentation topics and the mixture of presentation lengths (e.g. 30-min invited, 15-min offered and 5-min flash presentations). There were no negative responses regarding a lack of perceived diversity, or a negative impression of the diversity offered at the Symposium. The majority (15) of the diversity-coded responses were received as free-text responses to the statement ‘I feel the 2024 Education and Outreach Symposium was an inclusive environment’ indicating the link the respondents felt between the diversity offered by the Symposium and how they felt that was a part of generating an inclusive environment.

The generation of an inclusive environment is a key driver for the Education and Outreach Symposium. The successful delivery of this aim is supported by both the quantitative Likert-style answers and the qualitative free-text data, with seven responses coded as commenting positivity on the environment, with the majority (six) of these responses being received as free-text responses to the statement ‘I feel the 2024 Education and Outreach Symposium was an inclusive environment’. The theme of the environment was broken down into two aspects: positive atmosphere and room layout. This supports previous observations [4] that our work to generate an open and friendly atmosphere was a success, with two comments directly relating to this:

‘*I felt confident that my presence was welcomed*’

‘*[A] very friendly group*’

The decision to encourage interaction and discussion by using a cabaret-style seating arrangement appeared to be successful:

‘*[I] preferred [being] sat around tables as it was easier to have discussions with people*’

An unintended benefit of the seating arrangement was also highlighted in the responses:

‘*[it] made it easier for people to navigate the space*’

Although it is difficult to draw conclusions from a single comment, the choice of seating may have further enabled inclusivity by allowing attendees to move around more freely. This should be investigated in more detail in subsequent evaluations of the Symposium.

The final theme to be identified from the thematic content analysis was improvements, with seven responses coded. These contained a balanced number of responses in favour of longer (one response) or shorter (one response) presentation lengths. This, in conjunction with the positivity around the presentation length (three responses within the diversity theme), suggests that our chosen approach of a mixture of presentation durations was the optimal format. Five responses were coded to relate to improvements in scheduling, mostly noting that some of the flash talks occurred after the related poster session, or the request for more discussion sessions/time.

‘*I’d like there to be flash talks before the relevant poster session so that you can go straight to discuss with presenters*’

## Limitations

The data were collected from a single Symposium, with attendees being self-selecting. This study did not investigate the reasons why some individuals may have chosen to attend other parallel sessions. For instance, while participants responded positively to the cabaret-style seating, some potential attendees may have found this format unfamiliar or impractical. Further research could explore the factors influencing session choice and non-attendance.

## Author insights

The Education and Outreach Symposium has grown each year with more abstract submissions and delegates attending.

The Education and Outreach Symposium will run again in 2025. Based on our own reflections and feedback from participants, we plan to make the following changes to the programme:

- We plan to move all of the 5-min flash talks to the end of the first half-day session. This will be scheduled just before the poster session so that each presenter can ‘advertise’ their posters and provide a springboard for participants to engage with poster presenters. Three comments from the evaluation supported this.
- We are keen to engage the audience as active participants rather than passive ‘listeners’. One piece of feedback expressed a desire to ‘*hear people’s opinions on common issues*’. Therefore, for the next Symposium, we are planning a panel session to wrap up the end of the second half-day. The panel will have diverse representation of career stages and backgrounds (e.g. established academics, PhD student, recent graduate, undergraduate student). Participants will be able to ask the panel for their advice, thoughts or comments on a range of issues (e.g. advice for those new to teaching, career progression, etc.).
- The use of Padlet was well received to allow anonymous questions and comments to be posted, and we will be retaining this element.
- We used cabaret-style seating to increase engagement. This was mentioned in the feedback from participants and will be retained in future years.

At the 2023 Symposium, we became aware that some presenters had already submitted their work for publication, some were aiming to prepare manuscripts and others had not yet considered the impact of their work past their presentation or poster [4]. As a result of this, we obtained funding through the Microbiology Society’s Society-Supported Conference Grant scheme to run a pedagogic workshop that was designed for those who wanted to engage with microbiology/outreach pedagogy and did not know where to start and those who had started/completed a project but did not know how to write it up for publication. We delivered two events: one in-person and one online. Twenty-seven people participated across the two events. Feedback from participants indicated that they found the workshops informative and felt more confident to start writing up and disseminating their work. The workshops were sponsored by *Access Microbiology,* so look out for future publications in the Pedagogy Collection [3].

## Conclusions

As part of our ongoing commitment to making the Symposium welcoming and inclusive, we gathered both quantitative and qualitative feedback from conference delegates. This initiative marks a transition towards a more evidence-based method of assessing the Symposium’s impact and effectiveness.

### Research question 1 - inclusivity: does the Symposium effectively welcome a diverse audience across all career stages?

We included a range of strategies to increase inclusivity across its many facets. From supporting a diverse range of offered and invited talks, with a variety of talk lengths to facilitate the distribution of ideas from initial findings to fully publishable works, through to organizational planning regarding cabaret-style seating and anonymous questions via Padlet, the data collected supported the broad success of these. All questionnaire respondents felt that the Symposium was an inclusive environment, and free-text responses commented positively on the diversity of talks and the positivity of the environment.

### Research question 2 - relevance: is the content provided interesting to participants?

To encourage attendance at the Symposium, the topics covered had to be relevant to a wide range of delegates. The quantitative data fully supported our success in designing a relevant Symposium, as all respondents felt it was interesting. Specifically, mentioned in the free-text comments was the variety and diversity of talks.

It is likely that the focus on inclusivity and relevance alone is not driving the increase in abstract submission and attendance, but it is clear that the Symposium is growing in popularity.

We are now actively planning for the 2025 Symposium, using our own reflections and the delegate feedback to implement improvements. We will continue to work towards an inclusive, safe and relevant environment, both digitally and physically, for everybody in the microbiology education and outreach communities.

## Abbreviations

AI: artificial intelligence
AMR: antimicrobial resistance
EON: Education and Outreach Network
UN: United Nations
