# Erratum: Towards an inclusive conference experience: evaluation of the Education and Outreach Symposium at the Microbiology Society Annual Conference 2024

**DOI:** 10.1099/acmi.0.001048

**Published:** 2025-05-08

**Authors:** Melissa M. Lacey, Michael J. Dillon, Sean Goodman, Victoria Easton, Alison I. Graham

**Affiliations:** 1 School of Biosciences and Chemistry, Sheffield Hallam University, Sheffield, UK; 2 Peninsula Medical School, University of Plymouth, Plymouth, UK; 3 School of Dentistry, University of Liverpool, Liverpool, UK; 4 School of Molecular and Cellular Biology, University of Leeds, Leeds, UK; 5 School of Medicine, Newcastle University, Newcastle-upon-Tyne, UK

Due to a mistake in production this article was published with a wrong DOI.

The correct DOI is 10.1099/acmi.0.000995.v3.

Previous DOI was 10.1099/acmi.0.000947.v3.

The version of record was updated to reflect this correction.

We apologise for any inconvenience caused.

